# Inflecting Factors on Alzheimer's Disease Progression: The Interaction of Gut Microbiome, Oxidative Stress, and Nutritional Interventions

**DOI:** 10.2174/0115680266342624241127071044

**Published:** 2025-03-18

**Authors:** Melih Dagdeviren, Elif Bozcal

**Affiliations:** 1 Ege University, Faculty of Science, Department of Biology, General Biology Section, 35040, Bornova-İzmir, Turkiye;; 2 Ege University, Center for Drug Development and Pharmacokinetic Applications, 35100, Bornova-İzmir, Turkiye;; 3 Istanbul University, Faculty of Science, Department of Biology, Basic and Industrial Microbiology Section, 34134, Fatih-Istanbul, Turkiye

**Keywords:** Alzheimer’s Disease, Oxidative Stress, Gut Microbiota, Nutrition, Neuroinflammation, Gut-Brain Axis

## Abstract

Alzheimer's Disease (AD) is a complex neurological condition caused by various factors. Diet, oxidative stress, and the gut microbiota all play critical roles in the development of AD. Recent studies suggested a bidirectional relationship between the gut and the brain, emphasizing the pivotal role of the gut microbiome in influencing cognitive functions. For instance, dysbiosis, a disruption in the balance of gut microbial communities, has been linked to neuroinflammation and the accumulation of amyloid-beta plaques, hallmark features of AD. Oxidative stress, arising from an imbalance between free radicals and antioxidants, contributes significantly to AD pathology. The molecular mechanisms through which oxidative stress impacts neuronal health and exacerbates cognitive decline in AD patients are also relevant. Moreover, nutritional interventions emerge as promising strategies to modulate these inflecting factors. Dietary components, such as antioxidants, omega-3 fatty acids, and polyphenols, exhibit neuroprotective effects, potentially mitigating AD progression. In contrast, the Western diet has a high potential to abet AD onset. Mediterranean diet and/or intermittent fasting are more valuable diets that may help delay the AD onset or progression. Limitations like individual differences affect the efficacy of nutritional interventions. As a supporting therapy, personalized diets should be applied according to the patients’ special needs/microbiomes in the future. To gather current knowledge on the interconnected roles of the gut microbiome, oxidative stress, and nutritional interventions in AD is crucial. Understanding these interactions may pave the way for novel therapeutic approaches, as well as disputing the potential diets that can help improve AD patients' quality of life.

## 1 INTRODUCTION

Investigations conducted in the last decade showed that bacteria, fungi, parasites, and viruses coexist in the human intestinal environment, and microorganisms called microbiota live together with the human host in symbiosis [1]. The gut microbiota develops early in life and can be affected by genetic and environmental factors, as well as affecting brain health [2]. Neurological disorders such as Alzheimer`s Disease (AD), Parkinson’s Disease (PD), and Multiple Sclerosis (MS) involve progressive neuronal loss, yet their exact causes are unclear. The gut microbiome, considered the second human genome, plays a vital role in initiating or advancing these diseases through the gut-brain axis. Yet, the molecular processes by which the microbiome influences neurodegenerative diseases, particularly through immune modulation, are not well understood. Therefore, revealing disease initiation and the microbiome's impact on immune function in neurodegenerative diseases is important [3].

The gut-brain axis, crucial for physiological regulation, involves significant communication with the gut microbiota. Oxidative stress (OS) is a key pathogenic mechanism in neurodegenerative diseases and acute conditions. Specific microbiota types may increase inflammation and reactive oxygen species, promoting abnormal protein aggregation. Bidirectionally, brain lesions alter gut properties and microbiota. These insights are important to acknowledge new therapeutic options for neurological diseases [4].

OS is a critical factor in the onset and progression of various diseases, as supported by experimental and clinical studies. The release and toxicity of reactive oxygen and nitrogen species (ROS/RNS) are important. Cells produce unpaired electrons, generating ROS/RNS during normal processes or stress. At the same time, it is beneficial in controlled amounts for cell survival and pathogen degradation. Nevertheless, ROS/RNS create an imbalance with antioxidant defenses, and excess ROS/RNS can damage macromolecules (DNA, proteins, and lipids), affect multiple organs, and contribute to aging-related diseases like cancer, diabetes, and neurodegenerative conditions [5, 6]. Recent advances in aging research are improving treatments for age-related diseases and promoting a healthy, high-quality lifespan. Molecular and cellular damage, influenced by ROS, is believed to underlie age-associated alterations. ROS/RNS contribute to aging physiology and physiopathology, like genetic instability, shortening of telomeres, changes in gene expression, the decline in protein regulation, disrupted nutrient response, malfunctioning mitochondria, aging of cells, depletion of stem cells, altered intercellular communication [7]. The expression of antioxidant enzyme genes is suppressed because of epigenetic regulations in AD. This also fuels the OS and triggers neuronal death. Thus, antioxidant therapy is important in AD [8]. Aging is one of the major causes of imbalances in ROS/RNS metabolism and creates OS [9]. Not just ROS/RNS is important for neurodegenerative diseases like AD and PD. Also, iron species contribute to oxidative stress, generating harmful free radicals, especially the hydroxyl radical. The implications of OS and ferroptosis in AD are important. Strategies like iron chelation and antioxidant therapies to combat AD may create a new path [10].

“Alzheimer's Disease International” states that more than 50 million people are living with AD worldwide in 2020, and this number will increase to 152 million by 2050. Very few β-amyloid (Aβ) or tau-based approaches have proven effective in clinical studies [11]. AD involves multifactorial deterioration of neuronal networks, with abnormal protein accumulation contributing to its pathogenesis. Mitochondrial dysfunction, implicated in aging, increases AD risk. Dysfunctional mitochondria generate ROS, causing oxidative damage and amyloid progression. Amyloid-induced mitochondrial toxicity involves membrane permeability, calcium disruption, and altered oxidative phosphorylation. Inflammatory changes, particularly in amyloid-rich areas, contribute to neuronal dysfunction. Improved methods to visualize early neuroinflammation and agents modulating mitochondrial function are essential for monitoring and controlling AD therapeutic strategies [12].

Mitochondrial dysfunction produces ROS, then proteins are misfolded, and Aβ accumulates because of ROS-triggered proteasomal malfunctions. Besides, ROS activates microglia, which starts neuroinflammation. At the end of those processes, more troubled mitochondria arose, and AD progression accelerated. Also, neurons have a dramatically low number of catalase and glutathione when compared to other cells [13]. Under OS conditions, mitochondrial DNA fragments may be discarded into extracellular space and trigger inflammatory responses [14]. There are various pharmacological and nonpharmacological therapies targeting mitochondria, including antioxidants, diets, and mitochondrial transplantation [15]. Targeting mitochondria restoration for innovative drug discovery through the gut-brain axis has potential.

Biochemical alterations in the brain involving neuroinflammation, OS, and mitochondrial dysfunction are pivotal in mild cognitive impairment (MCI) and AD pathophysiology. A meta-analysis of human brain studies using ^1^H and ^31^P-Magnetig Resonance Spectroscopy reveals consistent alterations: decreased N-acetyl aspartate and creatine, increased myo-inositol in MCI and AD, decreased glutathione in MCI, and damaged energy homeostasis in AD. The hippocampus displays pronounced changes, suggesting its importance in early diagnosis and intervention monitoring. The targeting of energy metabolism associated with neuroinflammation/OS may be a beneficial effort for AD treatment strategies [16]. AD is a debilitating neurological disease without any cure, primarily affecting the aging population. Synaptic loss, correlated with cognitive decline, is an early mechanism in AD. OS, linked to aging and neurodegenerative diseases, impacts mitochondrial function, metal homeostasis, and antioxidant defense, affecting synaptic activity and neurotransmission. While ROS contributes to AD pathology, clinical trials of antioxidant therapies yield inconsistent results. Thus, innovative therapeutic strategies such as nutraceutical formulations are important [17]. Mitochondrial dysfunctions are recognized as key factors in gastroduodenal issues etiology, influencing pathogenesis rather than being mere consequences. OS triggers mitochondrial damage, generating molecular patterns that intensify inflammatory tissue damage. Mitochondrial dynamics, mitophagy, and interactions with gut pathogens contribute to gastrointestinal mucosal issues. Targeting mitochondria for innovative drug discovery against gastrointestinal disorders has potential [18].

Dietary antioxidants like omega-3 fatty acids and polyphenols neutralize ROS/RNS, improve membrane fluidity, and support synaptic function. Besides, it reduces cellular damage, neuroinflammation, Aβ plaques, and tau hyperphosphorylation. Thus, dietary antioxidants are crucial for AD [19, 20].

## 2 THE GUT-BRAIN AXIS AND THE GUT MICROBIOME

### 2.1 Microbiota-Gut-Brain Axis

The human microbiota, comprising bacteria, viruses, and fungi, plays a crucial role in health and significantly influences the immune, nervous, and gastrointestinal systems. Acquisition of these microbes, primarily in the gastrointestinal tract, shapes immune and neurological development. Factors like genetics, environment, diet, and drugs impact gut microbiota composition.

The term “Gut-Brain Axis” is very important for explaining the two-way communication between the Central Nervous System (CNS) and the Enteric Nervous System (ENS). This connection has a very important role throughout different life stages, from early development to old age, and affects various parts of the body, from the intestinal lumen to the CNS. Additionally, the “Gut-Brain Axis” has many functions, including immune, endocrine, nervous, and metabolic signals, and the ENS is located in the gastrointestinal system; it can regulate signals coming from sympathetic and parasympathetic systems, including the vagus nerve and prevertebral ganglia [1]. The gut microbiota is considered the “second brain” of the human body; therefore, it is significant for pathogen invasion, immune response, and host metabolism. Gut microbiota interacts with CNS *via* the called “microbiota-gut-brain axis” [21]. The “microbiota-gut-brain axis” is a two-way communication system that includes immune, neural, metabolic, and endocrine pathways, impacting brain development, neural function, and behavior. Recent studies indicate that an imbalance in gut microbiota may contribute not only to various immune and inflammatory diseases but also to neurological conditions like AD [22]. The CNS is protected by the blood-brain barrier (BBB), a semi-permeable membrane that regulates the exchange of molecules and nutrients between the bloodstream and the brain. However, as we age, the performance of this mechanism decreases, potentially allowing harmful intestinal microbiota and their products to enter the brain, which may lead to neurodegeneration and AD pathogenesis [21]. Gut microbiota can activate or stimulate neurons of the ENS, which may facilitate direct neural communication between the nerve, gut, and brain [23]. Bacterial products such as short-chain fatty acids (SCFAs) produced by the gut microbiota can have a neuromodulation effect and act directly on gastrointestinal cells by stimulating the synthesis of hormones such as leptin and ghrelin. Microbiota also plays a role in tryptophan metabolism; it can produce tryptophan catabolites as well as other metabolites, including neurotransmitters and hormones, which can be separated from the intestinal lumen and detected in the circulation to act as signaling molecules such as catecholamines and serotonin [23].

The bidirectional link between gut and brain activities *via* inflammatory signals. The gut-brain axis affects AD by promoting neuroinflammation, Aβ accumulation, and neurotransmitter imbalances due to gut dysbiosis. It also influences metabolic dysfunction, insulin resistance, and vagus nerve signaling, all of which contribute to cognitive decline and exacerbate disease progression [24].

### 2.2 Dysbiosis in Alzheimer's Disease

Dysbiosis, an imbalance in the gut microflora, is linked to neurological disorders. Exploring microbiome-host communication unveils therapeutic options. Preclinical and clinical studies on microbiome interventions, including probiotics, prebiotics, postbiotics, synbiotics, and fecal transplantation, showed promise in treating conditions like AD, PD, MS, autism, schizophrenia, epilepsy, and stroke [25]. Over 50 million people face cognitive impairment from neurodegenerative diseases like AD, PD, and stroke. Interactions among these conditions exacerbate the damage, as post-stroke dementia may induce further neurodegeneration. The gut microbiome, acting as an endocrine organ, produces bioactive metabolites affecting human physiology. Gut dysbiosis, linked to neurodegenerative and cerebrovascular diseases, accelerates cognitive decline. Factors like obesity, metabolic issues, cardiovascular diseases, sleep disorders, and inactivity contribute to dysbiosis, forming a harmful cycle in older individuals [26]. The human gut microbiome is linked to infection risk, and gut dysbiosis correlates with inflammatory responses affecting multiple organ systems. This connection between dysbiosis and age-related diseases involves communication with the intestinal mucosa and the immune system. Recent insights into the microbiome's role in aging open avenues for novel therapeutics aiming to shape a healthier microbiome, potentially preventing or treating age-related diseases [27].

BBB, crucial for central nervous system protection, faces paradoxical challenges in allowing harmful gut-derived factors into the brain. Dysbiosis-induced BBB permeability in mice prompts questions about how gut microbiota and its metabolites contribute to neuroinflammation, neurodegeneration, and brain aging. Chronic systemic inflammation from gut microbiota dysregulation may impair the BBB, affecting neuroinflammatory processes and aging, suggesting potential therapeutic targets [28].

Gut dysbiosis in AD increases gut permeability, allowing toxins like LPS and bacteria to enter the bloodstream and triggering systemic inflammation *via* toll-like receptor 4 (TLR4) pathways. This inflammation promotes neuroinflammation *via* cytokines, while microbial metabolites like LPS exacerbate Aβ plaque accumulation (microbiota-derived amyloid peptides can cross the BBB), worsening cognitive decline [29].

The gut microbiota acts as a crucial microbial endocrine organ, influencing human physiology, metabolism, and health. Alterations in microbial composition are linked to diseases like obesity, chronic kidney disease, cardiovascular diseases, inflammatory bowel diseases, non-alcoholic fatty liver disease, and more. The role of epigenetics (heritable DNA changes influenced by environmental factors such as nutrition, stress, and microbiome) in gene expression modifications is important. There are many studies investigating gut microbial function and its role in inflammatory and metabolic disorders [30]. The gut microbiota composition is dynamic and influenced by factors like diet, drugs, mucosa, the immune system, and microbiota itself. Natural variations can lead to dysbiosis, marked by decreased diversity and expansion of specific bacterial taxa. Mechanisms involve OS, bacteriophage induction, and toxin secretion, triggering rapid shifts in microbial groups. Intestinal dysbiosis is associated with various diseases, including inflammatory bowel diseases, obesity, and type II diabetes. Understanding changes leading to dysbiosis is crucial for comprehending the microbiota's impact on health and disease [31].

There is various evidence collected from different study subjects: primary human neural cells, mice studies, and human studies. Alteration in the gut microbiome has several outputs for AD progression. The same microbiome couldn’t be harmful, but with aging, these bacteria can secrete toxins that cause neuroinflammation. Liu *et al*. offer at least five lines of evidence for this issue, including antibiotic studies and hepatic encephalopathy in addition to the aforementioned ones [24, 32].

AD, which is marked by Aβ plaque and neurofibrillary tangle formation, lacks effective therapies. Current cerebrospinal fluid biomarkers include Aβ_1–42_, phosphorylated tau, and total tau, but the search for novel, accessible blood-based markers is ongoing. Potential biomarkers of AD include a variety of proteins, lipids, metabolites, OS-related molecules, and cytokines. Moreover, new markers such as miRNAs, lncRNAs, vitamins, and gut-microbiome-related molecules are emerging for diagnosing and tracking the progression of the disease beyond traditional markers [33].

Differentiated microbial diversity and microbial composition can be observed in AD patients compared to the control group. These observed changes may cause an inflammatory response in the intestine that can degrade the epithelial barrier. This may lead to increased translocation of proinflammatory products and an increase in bacterial products such as lipopolysaccharides (LPS), amyloids, DNA, proteins, and polysaccharides, which can cause autoimmunity. Systemic inflammation resulting from this condition can cause microglia activation, neuroinflammation, and BBB disorder [23, 34].

AD remains a complex neurodegenerative condition with an unclear pathogenesis. A study performed by a Chinese research group explored the connection between AD, gut microbiota dysbiosis, and the cholinergic anti-inflammatory pathway. Using Aβ_1–42_ injections in mice, researchers observed cognitive impairment, gut microbiota changes, and accelerated amyloidogenic pathways, linking CNS-induced dysbiosis to inhibited cholinergic anti-inflammatory pathways. These findings suggest a potential mechanism for gut-brain interactions in AD progression [35]. A group of scientists based in Italy investigated the association between gut microbiota-related products, systemic inflammation, and brain amyloidosis in AD. Analyzing metagenomic data from 89 individuals, the study revealed positive correlations between amyloidosis and bacterial products (LPS, acetate, valerate), pro-inflammatory cytokines, and endothelial dysfunction markers. Conversely, negative correlations are observed with butyrate and the anti-inflammatory cytokine IL10. The findings suggest that SCFAs and LPS may act as potential links between gut microbiota and AD pathology through systemic inflammation and endothelial dysfunction [36].

Neuronal communication in the CNS relies on intricate networks of synaptic proteins. Notably, neurexin-1, SNAP-25, synapsin-2, neuroligin, and SHANK3 are vital for efficient neurotransmission. Exposure to *Bacteroides fragilis* lipopolysaccharide (BF-LPS) in human neuronal-glial cell cultures leads to a significant downregulation of these proteins, mirroring findings in AD-affected brains. USA-based scientists explored the impact of gastrointestinal bacterial exudates on neurotoxicity, suggesting a link between BF-LPS exposure, altered immune responses, and cognitive deficits in the CNS [37]. Moreover, *Bacteroides* can contribute to AD *via* modulating microglia. It has been reported that some members of the *Bacteroidota* phylum are factors in AD pathogenesis as a result of repressing the phagocytic function of microglia, which causes impaired Aβ clearance and amyloid plaque accumulation [38].

Gastrointestinal microbiome-derived LPS has been found to target and encapsulate neuronal nuclei in AD brains, hindering genetic information flow. This action disrupts the expression of AD-related markers, including neurofilament light chain protein and synaptic phosphoprotein synapsin-1. The mechanism of LPS targeting and nuclear disruption remains unclear. Co-incubating LPS with Aβ_42_ peptide in human neuronal-glial cells supports a novel pathogenic role for Aβ_42_, potentially contributing to neuropathological deficits observed in AD by facilitating LPS entry into neurons and nuclear down-regulation of key components [39]. AD, a globally affecting form of dementia, is explored in a Turkiye-based study through metaproteomic analysis of gut microbiota. Using Nano-Liquid Chromatography-Mass Spectrometry, researchers compared proteomic shifts in early (3-month-old) and late (12-month-old) stages of AD in transgenic mice. Computational analysis revealed significant microbiota, proteome, and molecular changes in the intestine preceding disease symptoms. This research emphasizes the potential of identified phyla, proteins, and pathways as markers for early and late AD stages, urging further exploration of the intricate gut-brain axis in AD pathogenesis. They observed *Actinobacteria* and *Proteobacteria* as increasing targets, while *Firmicutes* was a decreasing target to look for [1]. Qu *et al*. evaluated the behavior and gut microbiota of AD model [TgCRND8 (Tg)] mice of different ages in a Morris water maze. Also, 16S rRNA sequencing was performed on microbiota. They reported that age-dependent gut dysbiosis, neuroinflammation response, Aβ accumulation, tau hyperphosphorylation, trimethylamine N-oxide (TMAO) overproduction, and cyclin-dependent kinase 5 (CDK5)/transcription 3 (STAT3) activation occurred in Tg mice. TMAO, a metabolite derived from gut microbes involved in choline metabolism, is a novel prognostic marker in cardiovascular diseases and has a potential role in various chronic diseases such as diabetes. Besides, the TMAO concentration significantly increased in the blood of mice with AD pathogenesis. They also noted that the aging deteriorated the gut microbiota composition of Tg mice, indicating potential intestinal inflammation and impaired gastrointestinal barrier integrity. Some pathogenic microbiota phyla, such as *Proteobacteria* and *Bacteroidetes,* are abundant in the gut and participate in the pro-inflammatory process, while the *Firmicutes* phylum and the *Lactobacillaceae* family showed anti-inflammatory activities. It has been reported that the relative abundance of *Lachnospiraceae, Bacteroidaceae, Veillonellaceae, Negativicutes*, and *Bacterodia* decreased significantly. In contrast, the relative abundance of *Ruminococcaceae, Lactobacillaceae*, *Actinobacteria,* and *Bacilli* increased in AD patients [40]. Zhang *et al*. evaluated the systemic changes and potential pathogenic risks in an endogenous melatonin reducer (EMR) mouse model lacking the arylalkylamine N-acetyltransferase gene, the key enzyme in melatonin synthesis, and discovered a new link between melatonin, AD, and the gut microbiota. Their findings indicated that EMR leads to systemic changes due to gut microbiota dysbiosis, which might contribute to AD and obesity. The study suggests that targeting the gut microbiota could be a potential strategy for preventing and treating those disorders [41].

Moreover, it has been noted that the intestinal microbiota and intestinal barrier function may be altered in older adults with normal cognition or a prodromal AD phenotype with subjective cognitive decline or amnestic MCI after certain orthopedic surgical procedures. Orthopedic surgery can induce gut microbiome dysbiosis in AD patients. Liu *et al*. have stated that surgery stimulates dysbiosis and intestinal barrier dysfunction in AD [42].

The connection between periodontitis, the BBB, and AD was known from clinical findings. As people get older and are continually exposed to pathogens, the BBB can become less effective, potentially leading to CNS pathology like AD. Bacteria associated with periodontitis have been connected to neuroinflammation and characteristics similar to AD. Researchers have found the pathogen DNA and virulence factors in the brains of AD patients. This suggests a possible link between periodontitis and the development of AD [43]. A study carried out in China explores how the salivary microbiota of individuals with periodontitis affect AD in transgenic mice. Salivae of people with periodontitis and healthy controls were gavaged into APPswe/PS1ΔE9 (PAP) mice, resulting in cognitive impairment, increased Aβ accumulation, and neuroinflammation after continuous application of periodontitis-related salivary. These AD-associated conditions correlated with gut microbial dysbiosis, intestinal inflammation, barrier impairment, and systemic inflammation, suggesting a link between periodontitis-related salivary microbiota and AD progression *via* the gut-brain axis. Periodontitis has a potential role in AD etiology and intervention strategies [44]. One of the links between infection and AD is edentulism, which is the complete loss of teeth. This condition may occur due to chronic periodontal disease due to *Enterococcus faecalis* (*E. faecalis*) infection. The capacity of *E. faecalis* to cause bacterial infection in rat cortical neurons and create early AD-like neurofibrillary epitopes was assessed. It has been suggested that there may be a link between intracellular alterations brought on by an *E. faecalis* infection and early AD-like neurofibrillary pathology [45].

In a multi-center study, the gut-brain axis and bile acid (BA) dysregulation in AD were investigated. Examining serum samples from 1464 individuals, including cognitively normal, early, and late MCI and AD cases, revealed lower levels of the “primary BA” (CA) and higher levels of “secondary BA” (DCA) in AD. DCA is produced by bacteria. The DCA: CA ratio, reflecting gut bacteria activity, is strongly correlated with cognitive decline. Genetic variants in immune response genes connected to AD are also linked to BA profiles, suggesting a relation between gut dysbiosis and AD pathogenesis [46].

Current findings indicate that gut microbiota, including *Actinobacteria*, *Bacteroidetes*, and *Firmicutes*, along with epigenetic processes, influence AD pathogenesis. The bidirectional interaction between gut microbiota and epigenetics is significant. Their dynamic nature and their potential to be targets for AD diagnosis/treatment are high. The complex interplay of these factors provides numerous opportunities to understand and address AD [47]. Changes in the diversity of the intestinal microbiota, as well as some genes, potentially lead to the progression of AD. In a study investigating the relationships of risky genetic variants with altered intestinal microbiota at the onset of AD, the Apolipoprotein ε4 allele and rs744373 have been identified as risk loci for AD among 12 genetic variants. In terms of microbial composition, *Proteobacteria*; the genera *Enterobacteriales, Deltaproteobacteria* and *Desulfovibrionales*, *Anaerofustis*, *Morganelia, Finegoldia,* and *Anaerotruncus* have been reported to be increased in AD subjects [48].

Dysbiosis affects mental health, with germ-free mice and antibiotics used to study gut microbiota-brain relationships. Concerns arise from limitations in these models, including alterations in the BBB. To address these, researchers treated adult mice with various antibiotics, finding that ampicillin, though orally bioavailable, did not enter the brain. Antibiotic-induced dysbiosis depleted bacteria-derived metabolites, altered lipid species, and affected cognitive behavior, particularly novel object recognition. Therefore, substances that can change gut microbiota composition, such as antibiotic agents, can affect the disease positively or negatively. Previous research indicates that antibiotic treatments can lead to changes in the gut microbiota.

Additionally, both animal and clinical studies have shown that antibiotic use and accompanying dysbiosis are associated with changes in behavior and brain chemistry [49, 50]. For example, it has been demonstrated that in APP/PS1 transgenic mice, antibiotic cocktail administration can increase cytokine levels and neuroinflammatory status and be associated with AD [51]. The study highlights the role of circulating metabolites and the neuropeptide Y system in cognitive impairment due to dysbiosis [52]. A clinical study performed in China compared microbiomes in AD (including amnestic MCI) and healthy controls. AD patients show decreased fecal microbial diversity, distinct composition, and altered taxa prevalence, notably a rise in *Enterobacteriaceae*. Correlations exist between microbiome abundance and clinical severity. Models based on predominant microbiota effectively distinguish AD and MCI from healthy controls, offering potential therapeutic insights and diagnostic markers [53].

Chronic noise exposure is associated with altered gut microbiome in both wild-type (WT) and early-onset AD (EOAD) mice, affecting bacterial-encoded functions related to metabolism, OS, and inflammation. Noise-induced irregularities in the gut-brain axis, including reduced tight junction proteins and elevated inflammatory mediators, contribute to worsened neuropathology in presymptomatic EOAD mice. This suggests that environmental noise, particularly in a genetic context, may exacerbate the progression of EOAD by disrupting the microbiome-gut-brain axis [54]. Noise can cause tau hyperphosphorylation and the formation of neurofibrillary tangles (NFT) and may play a crucial role in the development of AD by increasing Aβ and amyloid precursor protein (APP). Accumulation of NFT and Aβ can trigger synaptic malfunction and apoptosis of neurons, which can cause Alzheimer's dementia [55].

AD, the prevalent form of dementia, results from multifactorial causes, including genetic factors, age, and environmental toxins like air pollution (AP). AP exposure induces chronic oxidative stress in the brain, contributing to AD pathogenesis. While limited, epidemiological evidence supporting the AP-Oxidative Stress-AD link is crucial due to the significant prevalence of AP exposure and an aging population. Exploring the AP-OS-AD relationship is important for public health interventions [56].

Post-traumatic stress disorder (PTSD) should be addressed with an integrative biopsychological approach. It is essential to examine the impact of traumatic stress, changes in the brain, genetics, mitochondrial functioning, and immune regulation, drawing on diverse fields such as clinical psychology, neuroscience, genetics, and gut microbiota research. PTSD is also associated with microbiome and neurological disorders [57].

In AD, local Aβ_42_ deposits induce neuroinflammation, but gut microbiota dysbiosis, a known risk factor for mental health diseases, can also trigger brain inflammation. Using a *Drosophila* AD model, *Enterobacteria* infection worsened disease progression by enhancing immune hemocyte recruitment to the brain, leading to TNF-JNK mediated neurodegeneration. Genetic depletion of hemocytes reduced neuroinflammation and mitigated neurodegeneration, emphasizing the crucial duty of gut-brain crosstalk in managing AD neurodegeneration [58].

In a meta-analysis, gut microbiota differences in the AD spectrum were examined. In 11 studies with 378 healthy controls and 427 AD spectrum patients, reduced microbiota diversity was observed in AD, not MCI. AD patients exhibited an altered abundance of *Proteobacteria*, *Bifidobacterium*, *Phascolarctobacterium*, *Firmicutes*, *Clostridiaceae*, *Lachnospiraceae*, and *Rikenellaceae* compared to healthy controls. *Alistipes* and *Bacteroides* showed country-specific effects. Clinical stages influenced abundance variations in *Clostridiaceae* and *Phascolarctobacterium*, highlighting altered microbiota in AD spectrum patients mediated by countries and clinical stages [59].

In AD and MCI, gut dysbiosis, assessed through a meta-analysis of 17 studies (679 AD/MCI, 632 controls), reveals decreased species richness in AD microbiomes. *Bacteroides* abundance is higher in US cohorts and lower in Chinese cohorts, indicating region-specific changes. *Phascolarctobacterium* increases significantly in MCI, suggesting dysbiosis initiation in the prodromal stage. Diet and lifestyle relevance in AD pathophysiology is emphasized, and microbiome studies may aid early diagnosis and intervention in neurodegenerative disorders [60].

Gut fungal dysbiosis in AD patients was explored in a study involving 88 Chinese AD patients and 65 healthy controls. Although fungal diversity remained unchanged, taxonomic composition differed, revealing the enrichment of *Candida tropicalis* and *Schizophyllum commune* and a decrease in *Rhodotorula mucilaginosa* in AD patients. Interestingly, *C. tropicalis* and *S. commune* correlated positively with immune markers IP-10 and TNF-a, while *C. tropicalis* correlated negatively with IL-8 and IFN-g. The study sheds light on the intricate relationship between gut fungal dysbiosis and host immunity in AD, offering potential diagnostic and therapeutic potential [61].

The same group also investigated the gut microbiota alterations in 100 Chinese AD patients compared to 71 controls. AD patients exhibited reduced bacterial diversity and changes in taxonomic composition. Notably, butyrate-producing genera like *Faecalibacterium* decreased, correlating positively with cognitive scores, while lactate-producing genera like *Bifidobacterium* increased, showing an inverse correlation. This shift in gut dysbiosis may serve as a non-invasive biomarker for AD diagnosis. Predicted functional changes in fatty acid metabolism suggest the potential for developing personalized probiotics for Chinese AD patients [62].

Chronic alcohol consumption influences AD risk, with potential connections to gut microbiota dysbiosis and intestinal barrier dysfunction. In a study, ethanol was administered to a transgenic AD mouse model, observing changes in microbiota, barrier permeability, systemic inflammation, behavior, and AD pathology. While alcohol-induced dysbiosis and barrier changes, it did not worsen AD-like pathology or behavior, possibly due to the absence of systemic inflammation. Notable differences were observed based on genotype and sex [63].

Another controlled study investigated neuropathological constipation in Tg2576 transgenic mice for AD and its association with fecal microbiota dysbiosis. Tg2576 mice exhibited prominent constipation phenotypes, including altered stool parameters and colon length. Fecal microbiota analysis revealed shifts in *Bacteroidetes*, *Firmicutes*, and *Proteobacteria* populations. Fecal microbiota transplantation from Tg2576 to wild-type mice induced significant decreases in stool parameters and compromised enteric nervous system function, suggesting a tight link between neuropathological constipation in Tg2576 mice and fecal microbiota dysbiosis [64].

AD progression in 3×Tg-AD mice at different stages was investigated. Spatial memory deficits, Aβ accumulation, and gut microbiota alterations were assessed in three age groups: asymptomatic (3 m), presymptomatic (6 m), and symptomatic (9 m) stages. Results show spatial memory issues, Aβ accumulation, and weight gain starting at 6 months. Gut bacterial changes, specifically in *Desulfobacterota* and *Actinobacteriota*, at 6 months precede apparent memory deficits, suggesting gut microbiota alterations as an early mechanism in AD pathology [65].

AD has unclear etiology and no effective treatments. The significance of the gut microbiota in AD has become popular because of the “microbiota-gut-brain axis.” Gut dysbiosis, influenced by aging, environment, and lifestyle, can lead to neuroinflammation and contribute to AD neuropathology. The seed and soil model of neurocognitive disorders proposes a genetic predisposition (“seed”) and dysbiotic factors (“soil”) as conditions for AD development. Lifestyle modifications, like dietary changes, may mitigate dysbiosis and decrease the brain's vulnerability to AD. Neuroimaging and early interventions align with this model, emphasizing the microbiota-gut-brain axis's causal role in AD onset [66].

The microbiota-gut-brain axis enables two-way communication between the associated organs through metabolic, endocrine, neural, and immune pathways. Gut dysbiosis, linked to neuropsychiatric disorders, may contribute to AD pathogenesis by promoting Aβ aggregation, neuroinflammation, oxidative stress, and insulin resistance. It is possible to suggest potential therapeutic strategies by modifying gut microbiota composition for AD prevention and treatment [32].

## 3 NUTRITIONAL INTERVENTION AND THERAPEUTIC STRATEGIES

### 3.1 Potential Therapeutic Strategies for Neuroprotection

Recent treatments, such as anti-Aβ monoclonal antibodies approved by the Food and Drug Administration, have been shown to slow the progression of AD in patients. Recently, through research, gut microbiota has been shown to regulate multiple neurophysiological responses through connections between the autonomic nervous system, the gut-microbiome-brain axis, and the immune system [23]. Also, for PD therapy, α synuclein inhibition *via* small molecules on *Caenorhabditis elegans* has promising effects [67]. Similar approaches also have the potential as neuroprotective strategies for AD.

Gut dysbiosis, marked by an imbalance in pathobionts and symbionts, contributes to central nervous system disorders. Gastrointestinal symptoms often precede neurological symptoms in neurodegenerative diseases. Dysbiosis disrupts the intestinal barrier, allowing toxic substances into circulation, triggering chronic neuroinflammation and proteinopathies, leading to neurodegeneration. Therapeutic approaches, including pre/pro/synbiotics, dietary interventions, and fecal microbial transplantation, aim to modulate gut dysbiosis for neurodegenerative disease management [68]. There is a link between dysbiosis and cognitive-behavioral changes like cognitive impairment, dementia, and various behaviors such as depression, schizophrenia, and addiction. To elucidate the cognitive-behavioral correlates of dysbiosis, molecular aspects should be investigated, and solutions to the pathogenesis of neuropsychiatric disorders and potential therapeutic strategies should be provided [69].

### 3.2 Nutritional Intervention and Lifestyle for Neuroprotection

The Western diet (WD), high in saturated (SFA) and trans fatty acids (TFA), omega-6 polyunsaturated fatty acids (PUFA), and cholesterol, is linked to metabolic disorders like obesity and diabetes type 2, as well as brain disorders. Neuroinflammation, influenced by dietary fatty acids, plays a role, with diets rich in n-6 PUFA, SFA, and TFA increasing inflammation, while those with monounsaturated fatty acids, omega-3 PUFA, and sphingolipids decrease it. The complex relationship involves peripheral metabolism, adipose tissue, microbiome, intestine, and vasculature. The impact of nutrition on brain health and lipid-mediated signaling pathways is vital [70]. The types of food WD include increased inflammation, OS, and gut dysbiosis. Mechanisms like neuroinflammation, BBB dysfunction, mitochondrial dysfunction, and OS arose because WD contributes to AD progression. However, the Mediterranean diet, Ketogenic diet, and intermittent fasting give better results for improving gut microbiota and slower AD progression [71, 72].

A controlled study investigated the impact of a WD on cognitive function in mice. WD consumption led to reduced hippocampal synaptic plasticity, decreased brain-derived neurotrophic factor, activated ERK1/2, and postsynaptic damage. WD induced inflammatory signaling in the brain, ileum, liver, adipose tissue, and spleen, along with microglia stimulation. Additionally, it caused dysbiosis, dysregulated bile acid synthesis, and systemic inflammation, contributing to reduced neuroplasticity. The findings highlight the intricate links between WD, inflammation, and cognitive health [73].

There are several nutritional interventions and diets have shown promise in clinical trials for improving cognitive function and slowing AD progression. Thus there are both clinical and experimental studies are available to reveal the mechanism behind the gut-brain axis and AD [71, 72, 74].

The microbiome's impact on gastrointestinal and nervous systems through the gut-brain axis has prompted interest in microbiome modulation as a therapeutic approach. This is particularly relevant for neurodegenerative disorders like AD, which involves neuronal depletion. Probiotic, prebiotic, and synbiotic supplementation is suggested to alleviate AD symptoms and improve associated biomarkers by influencing inflammation, antioxidant properties, cognition, and metabolic activity. There is a potential for these approaches as novel prophylactics for AD treatment [75].

Precision Nutrition (PN) aims to tailor dietary recommendations based on individual circumstances and biological characteristics. A workshop convened by the NIH discussed the complexities involved in understanding and implementing PN. Key areas included genetics, physiology, and factors influencing dietary responses, such as baseline nutritional status, circadian rhythm, and environmental exposures. The workshop emphasized the need for systems approaches, new technologies, and workforce development to advance PN. The conclusion highlighted the necessity for thorough research considering diverse variables before achieving more precise nutrition recommendations [76].

Natural compounds in fruits and vegetables, acting as antioxidants, show promise in preventing diseases like AD. The effectiveness of antioxidant supplements is still debated, but a diet rich in antioxidants can effectively reduce damage due to OS, which also occurs in AD [6, 19].

Plant-derived polyphenols, found in dietary sources, show the potential to improve health and manage diseases by influencing gene expression. However, the safe and effective use of these bioactive compounds raises controversies. The lack of clinical data on dosage, formulations, bioavailability, genetic variations, gut microflora, and potential interactions complicates their application. It is important to investigate the molecular basis of epigenetic regulation affected by polyphenols and understand their effects on human health [77].

The gut microbiota functions as an extension of the human genome, serving as a vital metabolic interface between dietary components and gastrointestinal substances. Recent bacterial genome sequencing technology has revealed nutrients/nutraceuticals as key factors shaping the gut microbiome, influencing human health across metabolism, gastrointestinal physiology, nutrition, and immune function. The gut microbiome has a significant impact on the host’s metabolism and susceptibility to chronic diseases. Nutraceuticals may offer a means to manipulate the microbiome for defensive or therapeutic benefits [78].

A robust gut microbiota is crucial for overall health, influencing microbial balance, body growth, and immunity. Dietary plants and their bioactive phytochemicals shape microbiota, fostering health benefits. A bioactive phytochemical-rich diet promotes probiotic bacteria, hampering harmful residents. Also, these phytochemicals interact with microbiota; they transform, enhance bioactivity, and reduce toxicity [79]. Patchouli alcohol (PA), derived from Pogostemonis Herba, exhibits neuroprotective properties. A study conducted on the TgCRND8 transgenic AD mouse model explores PA's therapeutic effects and mechanisms. PA treatment improves daily living activities, mitigates cognitive deficits, and modulates amyloid precursor protein processing. It reduces Aβ levels, inhibits tau hyperphosphorylation, and relieves neuroinflammation. PA also restores gut dysbiosis and inhibits the C/EBPβ/AEP signaling pathway. Findings suggest PA as a promising treatment for AD, addressing cognitive deficits, Aβ plaques, tau pathology, and gut dysbiosis [80].

In recent years, gut dysbiosis has been increasingly linked to AD progression. In AD mouse models, changes in gut microbiota composition result in peripheral accumulation of phenylalanine and isoleucine, promoting pro-inflammatory T helper 1 cells. These infiltrate the brain, activating M1 microglia and contributing to AD-related neuroinflammation. Similar findings are observed in patients with MCI also. GV-971, a sodium oligomannate, mitigates gut dysbiosis and associated neuroinflammation, restoring behavioral abilities, suggesting a novel AD therapy *via* gut microbiota remodeling [81].

AD involves complex pathogenesis with inflammation, Aβ plaques, and neurofibrillary tangles contributing to neurodegeneration. Previous treatments targeting these factors have been unsuccessful. Recent studies indicated a connection between changes in the intestinal environment and gut microbiota *via* the gut-brain axis, potentially influencing AD pathogenesis. Remodeling gut microbiota for balance emerges as a novel therapeutic strategy for AD [82].

Two studies released by a China-based group consecutively tried to exhibit the initiation of microbe exposure and AD interaction in mouse models. Psychobiotics, such as *Bifidobacterium breve* CCFM1025 and WX, demonstrate neuroprotective effects in AD by improving behavioral abnormalities and modulating gut dysbiosis in mice. Treatment enhances synaptic plasticity and increases brain-derived neurotrophic factor (BDNF), fibronectin type III domain-containing protein 5 (FNDC5), and postsynaptic density protein 95 (PSD-95) concentrations. Certain bacterial taxa and their metabolites correlate with AD-related behaviors, indicating a gut-brain axis role in AD pathophysiology. This evidence suggests that *B.* breve CCFM1025 and WX can be novel probiotic interventions that delay AD progression [83].

Combining environmental enrichment training with *Bifidobacterium breve* CCFM1025 treatment effectively counteracts Aβ-induced cognitive impairment and reduces neuroinflammation in mice. This approach also restores gut microbiota dysbiosis and reverses microbial metabolite alterations associated with AD. The study suggests a promising multidomain intervention strategy, integrating dietary microbiome-based approaches and lifestyle changes, to stop cognitive deterioration and halt the advancement of AD [84].

Dysbiosis in AD can be addressed by oral bacteriotherapy, specifically SLAB51 probiotic formulation, offering a potential strategy to delay AD onset and progression. The study investigates the link between probiotic consumption and cerebral benefits in 3×Tg-AD and wild-type mice. Chronic supplementation with SLAB51 enhances cerebral expression of hypoxia-inducible factor-1α (HIF-1α), counters nitric oxide levels, and exhibits neuroprotective and anti-inflammatory effects, providing a promising option for AD prevention and treatment [85].

In a proof-of-concept trial, a precision medicine approach was applied to 25 patients with dementia or MCI, assessing multiple contributors to cognitive decline. Over nine months, a personalized protocol targeting inflammation, infection, dysbiosis, and other factors showed statistically significant improvement in cognitive measures, including MoCA scores and neurocognitive index. Besides, no serious adverse events were reported [86]. A novel honokiol (an extract of *Magnolia*) nanoscale drug delivery system (Nano-HO) with improved solubility and bioavailability was designed to enhance the neuroprotective effects against AD. In TgCRND8 mice, Nano-HO, compared to free HO, significantly improved cognitive deficits, reduced neuroinflammation, inhibited Aβ accumulation and tau hyperphosphorylation also prevented gut microbiota dysbiosis. The findings proposed that Nano-HO has strong potential as a therapeutic agent for AD treatment, surpassing the efficacy of free HO in multiple aspects [87].

**Fig. (1):**
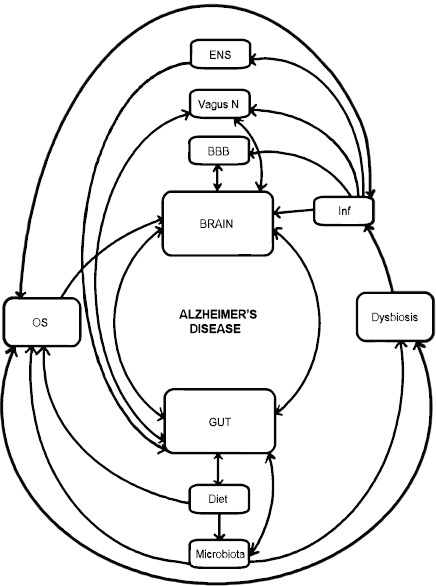
A simplified overview diagram of gut-brain axis and other concepts affected and/or affecting AD during its onset and prognosis. **Abbreviations:** Inf: inflammation, BBB: blood brain barrier, OS: oxidative stress, ENS: enteric nervous system, Vagus N: vagus nerve.

Gut dysbiosis and OS, triggered by a WD, start cascades of neuroinflammation, accelerating AD progression (Fig. **1**). Interventions like probiotics, antioxidants, and healthy diets potentially help patients improve their quality of life [24, 29]. Also, other healthy nutritional interventions are Mediterranean, Vegetarian, Ketogenic diets and intermittent fasting [72].

Probiotics, prebiotics, synbiotics, fecal microbiota transplantation, antioxidant supplementation, and diets rich in fiber, polyphenols, and omega-3 fatty acids can promote healthy gut microbiota and reduce OS [88, 89].

The human gut, essential for digestion and nutrient absorption, houses trillions of microbes influencing health. While the gut microbiome is implicated in various diseases, microbiome transplants emerge as a potential remedy. The impact of the intestines on diseases is known, and microbiome transplants are an option for treatment. It has potential benefits in age-related neurodegenerative disorders like PD and AD [90].

It is possible to mention the potential of ketogenic approaches to benefit cognition in healthy individuals, MCI, and AD. Cognitive functions, vital for daily activities, can decline due to aging or diseases like AD. Ketone bodies, an alternate fuel source, may compensate for impaired glucose metabolism, offering cognitive benefits. The neurophysiological changes observed through neuroimaging provide insights beyond molecular explanations, suggesting ketogenic strategies as a promising option for managing cognitive deterioration [91].

Urolithin A (UA), a gut-microbial metabolite, exhibits neuroprotective effects in the APP/PS1 mouse model of AD. UA improves cognitive function, prevents neuronal apoptosis, and enhances neurogenesis. It also reduces Aβ accumulation, peri-plaque microgliosis, and astrocytosis in the cortex and hippocampus. UA modulates critical signaling pathways, activating AMPK, inhibiting P65NF-κB and P38MAPK, and suppressing Bace1 and APP degradation. These findings suggest UA is a promising therapeutic drug for AD, providing cognitive protection through anti-inflammatory signaling [92].

Probiotics *Lactobacillus mucosae* NK41 and *Bifidobacterium longum* NK46, known for suppressing gut inflammation, demonstrate cognitive improvement in 5xFAD-transgenic mice. The probiotics reduce cognitive impairment, hippocampal Aβ, and inflammation markers while increasing BDNF expression. They also modulate gut microbiota composition, decreasing TNF-α-producing bacteria. A combined NK41 and NK46 formulation further enhances cognitive benefits, emphasizing the potential of probiotics in alleviating cognitive impairment and neuroinflammation by influencing gut bacteria and reducing LPS levels [93].

Probiotics Fermentation Technology (PFT), a kefir product, demonstrates efficacy in alleviating AD symptoms in a streptozotocin-induced AD mouse model. Administered orally for three weeks, PFT mitigates neuronal degeneration, restores acetylcholine levels, and improves cognition dose-dependently. PFT also reduces oxidative damage, inflammation, apoptosis, tau hyperphosphorylation, and Aβ plaque accumulation, comparable to the effects of simvastatin. The study suggests PFT's potential to regulate the gut microbiota and inhibit AD-related pathological events, offering a promising therapeutic option [94].

Tong *et al*. reported that an intestinal supplement, “Synbiotics” therapeutically affects gut microbiota and activates the Peroxisome proliferator-activated receptors (PPARs) pathway to inhibit AD progression. It has been stated that synbiotic treatment can significantly reduce neuroinflammation in the brains of APP/PS1 mice. It is emphasized that synbiotic treatment can effectively reduce the neuro-inflammatory response to delay the development of AD through the regulation of intestinal microflora [11]. Recent studies indicate that balancing intestinal dysbiosis with selective bioflavonoids is a very important solution to alleviate the activation of inflammasomes (which contribute to neuroinflammation) and stop the pathogenesis of AD. For example, two bioflavonoids called Epigallocatechin-3-gallate (EGCG) and genistein (GS) may emerge as a possible new treatment paradigm for maintaining healthy intestinal microbiota in AD due to their effects in modulating important AD signaling pathways. It can be emphasized that the combination of EGCG and GS may have a higher impact than either agent alone in weakening the signaling pathways associated with AD progression [95].

The lack of effective treatments for neurodegenerative disorders prompts a reevaluation of existing paradigms. From a systemic neurometabolic perspective, diseases such as ALS, PD, MS, and AD have common pathological features. Mitochondrial dysfunction associated with oxidative stress is identified as a very important factor alongside risk factors such as genetics, epigenetics, and environment. Restoring mitochondrial metabolism is important for potential therapeutic interventions in neurodegenerative disorders [96].

Mitochondrial dysfunction and mitophagy are important cellular malfunctions for AD. There are various pharmacological approaches targeting mitophagy [97]. From the point of view of microbiota knowledge, nonpharmacological approaches or nutritional interventions, such as caloric restriction, intermittent fasting, exercise, and diets rich in fiber and natural compounds, have advantages like less or no side effects, quick responses, supporting overall health and cost efficiency [98].

Dietary modifications can effectively alter gut microbiota composition and reduce OS in AD and PD patients, potentially improving their quality of life. Diets rich in fiber, polyphenols, and omega-3 fatty acids promote a healthy gut microbiome and reduce mechanisms fueling neurodegeneration [72].

## 4 DISCUSSION

Imbalances in the gut microbiota, or dysbiosis, contribute to autoimmune and neurodegenerative diseases like MS, ALS, Huntington's disease, PD, and AD by promoting inflammation, disrupting immune function, and altering neurochemical signaling. Dysbiosis can lead to increased intestinal permeability, allowing harmful substances to enter the bloodstream and trigger systemic inflammation and neuroinflammation, which are key factors in these diseases [99].

The gut-brain axis consists of a complex network of neural, immune, and endocrine pathways. Translating current knowledge faces several challenges and limitations; *i*) among individuals, gut microbiota composition varies; *ii*) mechanisms by which gut microbiota influence AD is not fully understood, *iii*) identifying OS early in the disease progression is very hard, *iv*) antioxidant interventions are limited, *v*) among individuals variability in nutrient absorption and metabolism, *vi*) food toxins [24, 88, 100, 101].

Most of the challenges have been known for several years and are mentioned in the sections above. However, some food toxins can alter gut microbiota and cause neuroinflammation. Also, they can penetrate through BBB by itself and act as a stressor on the CNS. Ochratoxin A (OTA) toxicity has been known for decades, but its potential to affect AD prognosis *via* gut dysbiosis is interesting.

Some risk factors, especially in the type of diet, can be eliminated by changing. However, some toxins can be consumed involuntarily. Being aware of these types of toxins, especially those initiated with food and/or water, is crucial. OTA, a toxic metabolite produced by *Aspergillus* and *Penicillium* fungi, is frequently present in unprocessed plant materials and animal feed. OTA induces OS, leading to genotoxicity and carcinogenicity, with the kidneys being the primary target organ. Excretion can occur through feces, urine, and breast milk. Changes in the Phe moiety affect cellular metabolism. Biological detoxification through microorganisms reduces DNA damage and cytotoxic effects. Antioxidants and dietary components show prophylactic potential against OTA toxicity in chronic exposure. Molecular changes caused by OTA may be linked to AD. In some studies, it has been observed that higher OTA levels in patients correlate with neurogenetic disorders [102]. The Phe moiety of OTA interacts with phenylalanine-handling proteins, which may cause impaired ribosomal protein expression. Thus, impaired mitochondrial function arose, and various catabolic pathways could be triggered [103].

The predominant exposure route for humans to OTA is through orally eaten meals and liquids. OTA contamination is quite common; it has been identified in numerous types of food and liquids consumed daily by individuals [104, 105].

Using food additives like baking soda and sugars during food processing can help reduce OTA levels. Also, consuming probiotics and synbiotics can reduce OTA concentrations in the gastrointestinal tract [106, 107].

*Lactobacillus acidophilus* is a very effective microorganism in converting OTA to a non-toxic form. Also, there are various ruminal microflora members that are valid for detoxification by OTA. Yeasts like *Saccharomyces cerevisiae* can detoxify OTA. Biological detoxification agents are environmentally friendly and sustainable in reducing OTA contamination in the gut. These agents are effective in mitigating OTA's genotoxic and cytotoxic effects; they mostly depend on the Phe moiety removal of OTA [108].

At the cellular level, OTA induces OS through phase I and II enzyme systems. Then ROS is generated, and glutathione is depleted in the cells [109]. Genotoxic and carcinogenic effects of OTA arose from DNA adduct formation, chromosomal aberration, and DNA single-strand breaking mechanisms [110]. OTA creates necrosis and apoptosis in kidneys *via* OS and mitochondrial dysfunction [111]. In rodents, OTA can actively cross BBB, potentially create OS in the brain, and trigger mechanisms that also lay the background of AD onset [112].

Several antioxidants and dietary components have defense and prophylactic potential against OTA toxicity by mitigating its effects on OS, DNA damage, and cytotoxicity. Curcumin, vitamins, polyphenols, zinc, and selenium are some supplements not just useful for detoxifying OTA but also helpful for ameliorating AD onset and prognosis [113, 114].

OTA exposure decreased the BDNF expression, which is similar to AD. Research on animal models has shown that OTA exposure leads to cognitive deficits, OS, and neuropathological changes similar to those observed in AD. *In vitro* studies have demonstrated that OTA induces apoptosis, necrosis, and other cytotoxic effects in neuronal cells, activating similar mechanisms in AD pathogenesis [112].

Most likely, the higher levels of OTA create genetic modifications, DNA damage, and single-strand breaks *via* OS and mitochondrial dysfunction. If those types of stressors or repetitive exposures cumulatively accumulate on nervous tissue, cell loss starts [102, 115].

Current strategies for treating gut microbiota disruptions include probiotic intake and fecal microbiota transplantation. Probiotics help restore healthy microbial balance, reducing inflammation and improving gut barrier function. Fecal microbiota transplantation involves transferring stool from a healthy donor to a patient, effectively resetting their gut microbiota. Both approaches have shown promise in alleviating symptoms and slowing disease progression, although more research is needed to optimize their efficacy [116].

For neurodegenerative diseases, therapeutic approaches might involve targeted dietary interventions, antioxidant therapy, and personalized microbiota modulation based on an individual's specific gut microbiome profile. Understanding the interactions between the gut microbiome, OS, and nutrition could lead to more precise treatments that slow neurodegeneration and improve the quality of life for patients with these conditions [88].

## CONCLUSION, CHALLENGES AND FUTURE DIRECTIONS

Advances in microbiota knowledge, particularly in biotechnology and molecular biology, have ushered in the “omics” era, transforming the understanding of physiological and pathophysiological processes. The microbiota-host relationship, influenced by genetics and environment, plays a vital role in health. Imbalances may contribute to autoimmune and neurodegenerative diseases like MS, ALS, Huntington's chorea, and AD. Treating gut microbiota disruptions through strategies like probiotic intake and stool transplantation shows promise in slowing neurodegeneration, offering hope for future therapeutic approaches [117].

Not just bacteria but also fungi from the intestines can cause neuroinflammation and autoimmune reactions during aging and AD development [118]. Mycobiota maintains gut health and adapts to environmental changes. Factors like diet, alcohol, and mycotoxins alter their composition, impacting immune function and contributing to complex human diseases [119]. In a human tissue study from Spain, it was shown that there are fungal cells in AD patients’ different brain regions when compared to healthy control tissues [120].

Understanding the interplay between genetics, environment, and gut microbiota can help develop personalized medicine approaches and microbiota-based therapies for complex human diseases. This knowledge is crucial for promoting health and preventing disease [121].

Various diets and supplements may help delay AD onset or progression by altering the gut microbiota. Limitations like individual differences, food toxins, and the degradation of antioxidant systems in patients’ brains affect the efficacy of nutritional interventions. Personalized diets or PN can be applied according to the patients’ special needs/microbiomes after the systems approaches, new technologies, and workforce developments are achieved in the future.

In conclusion, this focused topic allows for a deep dive into the specific interactions between the gut microbiome (Table **2**), OS, nutrition, and AD (Table **1**), providing a comprehensive overview of current research and potential options for future investigation and intervention.

## Figures and Tables

**Table 1:** Factors affecting Alzheimer's disease.

**Factor**		**References**
Oxidative Stress (OS)	Reactive Oxygen and Nitrogen Species (ROS/RNS)	[5, 6]
	Iron Species	[10]
Neuroinflammation		[16]
Neuroinflammation	Microglia Activation	[38]
Mitochondrial Dysfunction		[12]
Nutritional and Diet		[73]
Metabolites and Antibiotics		[49]
Gut-Brain Axis, Gut Microbiota, Dysbiosis	Short-Chain Fatty Acids (SCFAs)	[23]
	Lipopolysaccharides (LPS)	[29]
	Amyloids	[23]
	Deoxyribonucleic acid (DNA)	[5, 6]
	Proteins	[5, 6]
	Polysaccharides	[5, 6]
	Trimethylamine N-oxide (TMAO)	[40]
	Valerate	[36]
	Biogenic Amines (*e.g*., Serotonin)	[21]
Bile Acid Dysregulation		[46]
Epigenetic Processes		[47]
Traumatic Stress		[57]
Orthopedic Surgery		[42]
Lipid and Glucose Metabolism Alterations		[91]
Apolipoprotein ε4 allele and rs744373		[48]
Environmental Noise		[54]
Air Pollution		[56]

**Table 2:** Microorganisms affecting Alzheimer’s disease.

**Subject Organism**	**Taxonomic Nomenclature of Affecting Microorganisms**	**References**
Human neuronal-glial cells	*Bacteroides fragilis* (LPS)	[37]
-	*Bacteroides*	[38]
Mice	*Actinobacteria, Proteobacteria, Firmicutes*	[1]
-	*Proteobacteria, Bacteroidetes, Firmicutes, Lactobacillaceae, Lachnospiraceae, Bacteroidaceae, Veillonellaceae, Negativicutes, Bacterodia, Ruminococcaceae, Lactobacillaceae, Actinobacteria*	[40]
Rat cortical neurons	*Enterococcus faecalis*	[45]
-	*Actinobacteria, Bacteroidetes, Firmicutes*	[47]
AD Patients	*Enterobacteriales, Deltaproteobacteria* and *Desulfovibrionales*, *Anaerofustis*, *Morganelia, Finegoldia, Anaerotruncus*	[48]
AD and MCI Patients	*Enterobacteriaceae*	[53]
*Drosophila melanogaster*	*Enterobacteria*	[58]
AD Patients	*Proteobacteria, Bifidobacterium, Phascolarctobacterium, Firmicutes, Clostridiaceae, Lachnospiraceae, Rikenellaceae, Alistipes, Bacteroides*	[59]
AD and MCI Patients	*Bacteroides, Phascolarctobacterium*	[60]
AD Patients	*Candida tropicalis, Schizophyllum commune, Rhodotorula mucilaginosa*	[61]
AD Patients	*Faecalibacterium, Bifidobacterium*	[62]
Tg2576 Mice	*Bacteroidetes, Firmicutes, Proteobacteria*	[64]
3×Tg-AD Mice	*Desulfobacterota, Actinobacteriota*	[65]

## LIST OF ABBREVIATIONS

AD: Alzheimer's Disease
AP: Air Pollution
APP: Amyloid Precursor Protein
Aβ: β-Amyloid
BA: Bile Acid
BBB: Blood-Brain Barrier
BDNF: Brain-Derived Neurotrophic Factor
BF-LPS: *Bacteroides fragilis* Lipopolysaccharide
CSN: Central Nervous System
DNA: Deoxyribonucleic acid
E. faecalis: *Enterococcus faecalis*
EGCG: Epigallocatechin-3-gallate
EMR: Endogenous Melatonin Reducer
ENS: Enteric Nervous System
EOAD: Early-Onset AD
FNDC5: Fibronectin Type III Domain-Containing Protein 5
GS: Genistein
HIF-1α: Hypoxia-Inducible Factor-1α
MCI: Mild Cognitive Impairment
MS: Multiple Sclerosis
NFT: Neurofibrillary Tangles
OS: Oxidative Stress
OTA: Ochratoxin A
PA: Patchouli Alcohol
PD: Parkinson’s Disease
PFT: Probiotics Fermentation Technology
PN: Precision Nutrition
PPARs: Peroxisome Proliferator-Activated Receptors
PSD-95: Postsynaptic Density Protein 95
PTSD: Post-Traumatic Stress Disorder
PUFA: omega-6 polyunsaturated fatty acids
ROS/RNS: Reactive Oxygen Species/Reactive Nitrogen Species
SCFAs: Short-Chain Fatty Acids
TMAO: Trimethylamine N-oxide
UA: Urolithin A
WD: Western Diet

## References

[1] Ayan E., DeMirci H., Serdar M.A., Palermo F., Baykal A.T. (2023). Bridging the gap between gut microbiota and alzheimer’s disease: A metaproteomic approach for biomarker discovery in transgenic mice.. Int. J. Mol. Sci..

[2] Warren A., Nyavor Y., Zarabian N., Mahoney A., Frame L.A. (2024). The microbiota-gut-brain-immune interface in the pathogenesis of neuroinflammatory diseases: A narrative review of the emerging literature.. Front. Immunol..

[3] Cheng Y., Chen C., Zhang F. (2023). Immunity orchestrates a bridge in gut-brain axis of neurodegenerative diseases.. Ageing Res. Rev..

[4] Dumitrescu L., Popescu-Olaru I., Cozma L., Tulbă D., Hinescu M.E., Ceafalan L.C., Gherghiceanu M., Popescu B.O. (2018). Oxidative stress and the microbiota‐gut‐brain axis.. Oxid. Med. Cell. Longev..

[5] Hepel M., Andreescu S. (2015). Oxidative stress and human health..

[6] Thapa A., Carroll N. (2017). Dietary modulation of oxidative stress in alzheimer’s disease.. Int. J. Mol. Sci..

[7] Gil del Valle L. (2015). Oxidative stress: diagnostics, prevention, and therapy..

[8] Houldsworth A. (2023). Role of oxidative stress in neurodegenerative disorders: A review of reactive oxygen species and prevention by antioxidants.. Brain Commun..

[9] Yang J., Luo J., Tian X., Zhao Y., Li Y., Wu X. (2024). Progress in understanding oxidative stress, aging, and aging-related diseases.. Antioxidants.

[10] Zhao Z. (2019). Iron and oxidizing species in oxidative stress and alzheimer’s disease.. Aging Med. (Milton).

[11] Tong Y., Lu G., Guo J., Liu M., Dai Y., Zhang J., Xu X., Wang Z., Zhang G. (2024). A new intestinal supplement ‘synbiotics’ therapeutically regulates gut microbiota and activates ppars pathway to inhibit alzheimer’s disease progression in mouse models.. New Microbiol..

[12] Verri M., Pastoris O., Dossena M., Aquilani R., Guerriero F., Cuzzoni G., Venturini L., Ricevuti G., Bongiorno A.I. (2012). Mitochondrial alterations, oxidative stress and neuroinflammation in alzheimer's disease.. Int. J. Immunopathol. Pharmacol..

[13] Jurcău M.C., Andronie-Cioara F.L., Jurcău A., Marcu F., Ţiț D.M., Pașcalău N., Nistor-Cseppentö D.C. (2022). The link between oxidative stress, mitochondrial dysfunction and neuroinflammation in the pathophysiology of alzheimer’s disease: Therapeutic implications and future perspectives.. Antioxidants.

[14] Picca A., Calvani R., Coelho-Junior H.J., Landi F., Bernabei R., Marzetti E. (2020). Mitochondrial dysfunction, oxidative stress, and neuroinflammation: Intertwined roads to neurodegeneration.. Antioxidants.

[15] Sinha J.K., Jorwal K., Singh K.K., Han S.S., Bhaskar R., Ghosh S. (2024). The potential of mitochondrial therapeutics in the treatment of oxidative stress and inflammation in aging.. Mol. Neurobiol..

[16] Song T., Song X., Zhu C., Patrick R., Skurla M., Santangelo I., Green M., Harper D., Ren B., Forester B.P., Öngür D., Du F. (2021). Mitochondrial dysfunction, oxidative stress, neuroinflammation, and metabolic alterations in the progression of alzheimer’s disease: A meta-analysis of in vivo magnetic resonance spectroscopy studies.. Ageing Res. Rev..

[17] Tönnies E., Trushina E. (2017). Oxidative stress, synaptic dysfunction, and alzheimer’s disease.. J. Alzheimers Dis..

[18] Mazumder S., Bindu S., De R., Debsharma S., Pramanik S., Bandyopadhyay U. (2022). Emerging role of mitochondrial damps, aberrant mitochondrial dynamics and anomalous mitophagy in gut mucosal pathogenesis.. Life Sci..

[19] Essa M.M., Vijayan R.K., Castellano-Gonzalez G., Memon M.A., Braidy N., Guillemin G.J. (2012). Neuroprotective effect of natural products against alzheimer’s disease.. Neurochem. Res..

[20] Dyall S.C. (2015). Long-chain omega-3 fatty acids and the brain: A review of the independent and shared effects of epa, dpa and dha.. Front. Aging Neurosci..

[21] Liu S., Gao J., Liu K., Zhang H.L. (2021). Microbiota-gut-brain axis and alzheimer’s disease: Implications of the blood-brain barrier as an intervention target.. Mech. Ageing Dev..

[22] Chui Z.S.W., Chan L.M.L., Zhang E.W.H., Liang S., Choi E.P.H., Lok K.Y.W., Tun H.M., Kwok J.Y.Y. (2024). Effects of microbiome-based interventions on neurodegenerative diseases: A systematic review and meta-analysis.. Sci. Rep..

[23] Blivet G., Roman F.J., Lelouvier B., Ribière C., Touchon J. (2024). Photobiomodulation therapy: A novel therapeutic approach to alzheimer’s disease made possible by the evidence of a brain–gut interconnection.. J. Integr. Neurosci..

[24] Junyi L., Yueyang W., Bin L., Xiaohong D., Wenhui C., Ning Z., Hong Z. (2024). Gut microbiota mediates neuroinflammation in alzheimer’s disease: Unraveling key factors and mechanistic insights.. Mol. Neurobiol..

[25] Sorboni S.G., Moghaddam H.S., Jafarzadeh-Esfehani R., Soleimanpour S. (2022). A comprehensive review on the role of the gut microbiome in human neurological disorders.. Clin. Microbiol. Rev..

[26] Koszewicz M., Jaroch J., Brzecka A., Ejma M., Budrewicz S., Mikhaleva L.M., Muresanu C., Schield P., Somasundaram S.G., Kirkland C.E., Avila-Rodriguez M., Aliev G. (2021). Dysbiosis is one of the risk factor for stroke and cognitive impairment and potential target for treatment.. Pharmacol. Res..

[27] Haran J.P., McCormick B.A. (2021). Aging, frailty, and the microbiome—how dysbiosis influences human aging and disease.. Gastroenterology.

[28] Mou Y., Du Y., Zhou L., Yue J., Hu X., Liu Y., Chen S., Lin X., Zhang G., Xiao H., Dong B. (2022). Gut microbiota interact with the brain through systemic chronic inflammation: Implications on neuroinflammation, neurodegeneration, and aging.. Front. Immunol..

[29] Popescu C., Munteanu C., Anghelescu A., Ciobanu V., Spînu A., Andone I., Mandu M., Bistriceanu R., Băilă M., Postoiu R.L., Vlădulescu-Trandafir A.I., Giuvara S., Malaelea A.D., Onose G. (2024). Novelties on neuroinflammation in alzheimer’s disease–focus on gut and oral microbiota involvement.. Int. J. Mol. Sci..

[30] Srivastava S., Aditi P., Yadav D. (2023). The Metabolic Syndrome..

[31] Weiss G.A., Hennet T. (2017). Mechanisms and consequences of intestinal dysbiosis.. Cell. Mol. Life Sci..

[32] Liu S., Gao J., Zhu M., Liu K., Zhang H.L. (2020). Gut microbiota and dysbiosis in alzheimer’s disease: Implications for pathogenesis and treatment.. Mol. Neurobiol..

[33] Varesi A., Carrara A., Pires V.G., Floris V., Pierella E., Savioli G., Prasad S., Esposito C., Ricevuti G., Chirumbolo S., Pascale A. (2022). Blood-based biomarkers for alzheimer’s disease diagnosis and progression: An overview.. Cells.

[34] Kuwahara A., Matsuda K., Kuwahara Y., Asano S., Inui T., Marunaka Y. (2020). Microbiota-gut-brain axis: Enteroendocrine cells and the enteric nervous system form an interface between the microbiota and the central nervous system.. Biomed. Res..

[35] Qian X., Liu X., Chen G., Chen S., Tang H. (2022). Injection of amyloid-β to lateral ventricle induces gut microbiota dysbiosis in association with inhibition of cholinergic anti-inflammatory pathways in alzheimer’s disease.. J. Neuroinflammation.

[36] Marizzoni M., Cattaneo A., Mirabelli P., Festari C., Lopizzo N., Nicolosi V., Mombelli E., Mazzelli M., Luongo D., Naviglio D., Coppola L., Salvatore M., Frisoni G.B. (2020). Short-chain fatty acids and lipopolysaccharide as mediators between gut dysbiosis and amyloid pathology in alzheimer’s disease.. J. Alzheimers Dis..

[37] Zhao Y., Sharfman N.M., Jaber V.R., Lukiw W.J. (2019). Down-regulation of essential synaptic components by gi-tract microbiome-derived lipopolysaccharide (lps) in lps-treated human neuronal-glial (hng) cells in primary culture: Relevance to alzheimer’s disease (ad).. Front. Cell. Neurosci..

[38] Wasén C., Beauchamp L.C., Vincentini J., Li S., LeServe D.S., Gauthier C., Lopes J.R., Moreira T.G., Ekwudo M.N., Yin Z., da Silva P., Krishnan R.K., Butovsky O., Cox L.M., Weiner H.L. (2024). Bacteroidota inhibit microglia clearance of amyloid-beta and promote plaque deposition in alzheimer’s disease mouse models.. Nat. Commun..

[39] Lukiw W.J., Li W., Bond T., Zhao Y. (2019). Facilitation of gastrointestinal (gi) tract microbiome-derived lipopolysaccharide (lps) entry into human neurons by amyloid beta-42 (aβ42) peptide.. Front. Cell. Neurosci..

[40] Qu C., Xu Q.Q., Yang W., Zhong M., Yuan Q., Xian Y.F., Lin Z.X. (2023). Gut dysbiosis aggravates cognitive deficits, amyloid pathology and lipid metabolism dysregulation in a transgenic mouse model of alzheimer’s disease.. J. Pharm. Anal..

[41] Zhang B., Chen T., Cao M., Yuan C., Reiter R.J., Zhao Z., Zhao Y., Chen L., Fan W., Wang X., Zhou X., Li C. (2022). Gut microbiota dysbiosis induced by decreasing endogenous melatonin mediates the pathogenesis of alzheimer’s disease and obesity.. Front. Immunol..

[42] Liu F., Duan M., Fu H., Zhao G., Han Y., Lan F., Ahmed Z., Cao G., Li Z., Ma D., Wang T. (2022). Orthopedic surgery causes gut microbiome dysbiosis and intestinal barrier dysfunction in prodromal alzheimer disease patients.. Ann. Surg..

[43] Kouki M.A., Pritchard A.B., Alder J.E., Crean S. (2022). Do periodontal pathogens or associated virulence factors have a deleterious effect on the blood-brain barrier, contributing to alzheimer’s disease?. J. Alzheimers Dis..

[44] Lu J., Zhang S., Huang Y., Qian J., Tan B., Qian X., Zhuang J., Zou X., Li Y., Yan F. (2022). Periodontitis-related salivary microbiota aggravates alzheimer’s disease via gut-brain axis crosstalk.. Gut Microbes.

[45] Underly R., Song M.S., Dunbar G.L., Weaver C.L. (2016). Expression of alzheimer-type neurofibrillary epitopes in primary rat cortical neurons following infection with enterococcus faecalis.. Front. Aging Neurosci..

[46] MahmoudianDehkordi S., Arnold M., Nho K., Ahmad S., Jia W., Xie G., Louie G., Kueider-Paisley A., Moseley M. A., Thompson J. W., St John Williams L., Tenenbaum J. D., Blach C., Baillie R., Han X., Bhattacharyya S., Toledo J. B., Schafferer S., Klein S., Koal T., Risacher S. L., Allan Kling M., Motsinger-Reif A., Rotroff D. M., Jack J., Hankemeier T., Bennett D. A., De Jager P. L., Trojanowski J. Q., Shaw L. M., Weiner M. W., Doraiswamy P. M., van Duijn C. M., Saykin A. J., Kastenmüller G., Kaddurah-Daouk R. (2019). Altered bile acid profile associates with cognitive impairment in alzheimer’s disease—an emerging role for gut microbiome.. Alzheimers Dement..

[47] Nagu P., Parashar A., Behl T., Mehta V. (2021). Gut microbiota composition and epigenetic molecular changes connected to the pathogenesis of alzheimer’s disease.. J. Mol. Neurosci..

[48] Hou M., Xu G., Ran M., Luo W., Wang H. (2021). apoe-ε4 carrier status and gut microbiota dysbiosis in patients with alzheimer disease.. Front. Neurosci..

[49] Angelucci F., Cechova K., Amlerova J., Hort J. (2019). Antibiotics, gut microbiota, and alzheimer’s disease.. J. Neuroinflammation.

[50] Neuman H., Forsythe P., Uzan A., Avni O., Koren O. (2018). Antibiotics in early life: Dysbiosis and the damage done.. FEMS Microbiol. Rev..

[51] Minter M.R., Zhang C., Leone V., Ringus D.L., Zhang X., Oyler-Castrillo P., Musch M.W., Liao F., Ward J.F., Holtzman D.M., Chang E.B., Tanzi R.E., Sisodia S.S. (2016). Antibiotic-induced perturbations in gut microbial diversity influences neuro-inflammation and amyloidosis in a murine model of alzheimer’s disease.. Sci. Rep..

[52] Fröhlich E.E., Farzi A., Mayerhofer R., Reichmann F., Jačan A., Wagner B., Zinser E., Bordag N., Magnes C., Fröhlich E., Kashofer K., Gorkiewicz G., Holzer P. (2016). Cognitive impairment by antibiotic-induced gut dysbiosis: Analysis of gut microbiota-brain communication.. Brain Behav. Immun..

[53] Liu P., Wu L., Peng G., Han Y., Tang R., Ge J., Zhang L., Jia L., Yue S., Zhou K., Li L., Luo B., Wang B. (2019). Altered microbiomes distinguish alzheimer’s disease from amnestic mild cognitive impairment and health in a chinese cohort.. Brain Behav. Immun..

[54] Chi H., Cao W., Zhang M., Su D., Yang H., Li Z., Li C., She X., Wang K., Gao X., Ma K., Zheng P., Li X., Cui B. (2021). Environmental noise stress disturbs commensal microbiota homeostasis and induces oxi-inflammmation and ad-like neuropathology through epithelial barrier disruption in the eoad mouse model.. J. Neuroinflammation.

[55] Cui B., Li K. (2013). Chronic noise exposure and alzheimer disease: Is there an etiological association?. Med. Hypotheses.

[56] Moulton P.V., Yang W. (2012). Air pollution, oxidative stress, and alzheimer’s disease.. J. Environ. Public Health.

[57] Varadarajan S., Behnke A., Gumpp A.M., Mavioglu R.N., Fissler P., Kolassa I-T., Cloitre M. (2022). Evidence Based Treatments for Trauma-Related Psychological Disorders: A Practical Guide for Clinicians; Schnyder, U..

[58] Wu S.C., Cao Z.S., Chang K.M., Juang J.L. (2017). Intestinal microbial dysbiosis aggravates the progression of alzheimer’s disease in drosophila.. Nat. Commun..

[59] Hung C.C., Chang C.C., Huang C.W., Nouchi R., Cheng C.H. (2022). Gut microbiota in patients with alzheimer’s disease spectrum: A systematic review and meta-analysis.. Aging (Albany NY).

[60] Jemimah S., Chabib C.M.M., Hadjileontiadis L., AlShehhi A. (2023). Gut microbiome dysbiosis in alzheimer’s disease and mild cognitive impairment: A systematic review and meta-analysis.. PLoS One.

[61] Ling Z., Zhu M., Liu X., Shao L., Cheng Y., Yan X., Jiang R., Wu S. (2021). Fecal fungal dysbiosis in chinese patients with alzheimer’s disease.. Front. Cell Dev. Biol..

[62] Ling Z., Zhu M., Yan X., Cheng Y., Shao L., Liu X., Jiang R., Wu S. (2021). Structural and functional dysbiosis of fecal microbiota in chinese patients with alzheimer’s disease.. Front. Cell Dev. Biol..

[63] Frausto D.M., Engen P.A., Naqib A., Jackson A., Tran L., Green S.J., Shaikh M., Forsyth C.B., Keshavarzian A., Voigt R.M. (2022). Impact of alcohol-induced intestinal microbiota dysbiosis in a rodent model of alzheimer’s disease.. Front. Aging.

[64] Kim J.E., Roh Y.J., Choi Y.J., Lee S.J., Jin Y.J., Song H.J., Seol A.Y., Son H.J., Hong J.T., Hwang D.Y. (2022). Dysbiosis of fecal microbiota in tg2576 mice for alzheimer’s disease during pathological constipation.. Int. J. Mol. Sci..

[65] Wei Z., Li D., Shi J. (2023). Alterations of spatial memory and gut microbiota composition in alzheimer’s disease triple-transgenic mice at 3, 6, and 9 months of age.. Am. J. Alzheimers Dis. Other Demen..

[66] Hung C.C., Crowe-White K.M., McDonough I.M. (2023). A seed and soil model of gut dysbiosis in alzheimer’s disease.. Aging (Albany NY).

[67] Srivastava T., Tyagi D., Fatima S., Sathyan M.T.V., Raj R., Sharma A., Chaturvedi M., Sinha M., Shishodia S.K., Kumar D., Sharma S.K., Shankar J., Satish A., Priya S. (2024). A natural small molecule‐mediated inhibition of alpha‐synuclein aggregation leads to neuroprotection in caenorhabditis elegans.. J. Neurochem..

[68] Chidambaram S.B., Essa M.M., Rathipriya A.G., Bishir M., Ray B., Mahalakshmi A.M., Tousif A.H., Sakharkar M.K., Kashyap R.S., Friedland R.P., Monaghan T.M. (2022). Gut dysbiosis, defective autophagy and altered immune responses in neurodegenerative diseases: Tales of a vicious cycle.. Pharmacol. Ther..

[69] Luca M., Chattipakorn S.C., Sriwichaiin S., Luca A. (2020). Cognitive-behavioural correlates of dysbiosis: A review.. Int. J. Mol. Sci..

[70] (2022). Custers; Emma, E.M.; Kiliaan; Amanda, J. Dietary lipids from body to brain.. Prog. Lipid Res..

[71] Lou X. (2023). I.; Ali, K.; Chen, Q. Effect of nutrition in alzheimer’s disease: A systematic review.. Front. Neurosci..

[72] Ayten Ş., Bilici S. (2024). Modulation of gut microbiota through dietary intervention in neuroinflammation and alzheimer’s and parkinson’s diseases.. Curr. Nutr. Rep..

[73] Jena P.K., Sheng L., Di Lucente J., Jin L.W., Maezawa I., Wan Y.J.Y. (2018). Dysregulated bile acid synthesis and dysbiosis are implicated in western diet–induced systemic inflammation, microglial activation, and reduced neuroplasticity.. FASEB J..

[74] Power R., Nolan J.M., Prado-Cabrero A., Roche W., Coen R., Power T., Mulcahy R. (2022). Omega-3 fatty acid, carotenoid and vitamin e supplementation improves working memory in older adults: A randomised clinical trial.. Clin. Nutr..

[75] Arora K., Green M., Prakash S. (2020). The microbiome and alzheimer’s disease: Potential and limitations of prebiotic, synbiotic, and probiotic formulations.. Front. Bioeng. Biotechnol..

[76] Lee B.Y., Ordovás J.M., Parks E.J., Anderson C.A.M., Barabási A.L., Clinton S.K., de la Haye K., Duffy V.B., Franks P.W., Ginexi E.M., Hammond K.J., Hanlon E.C., Hittle M., Ho E., Horn A.L., Isaacson R.S., Mabry P.L., Malone S., Martin C.K., Mattei J., Meydani S.N., Nelson L.M., Neuhouser M.L., Parent B., Pronk N.P., Roche H.M., Saria S., Scheer F.A.J.L., Segal E., Sevick M.A., Spector T.D., Van Horn L., Varady K.A., Voruganti V.S., Martinez M.F. (2022). Research gaps and opportunities in precision nutrition: An nih workshop report.. Am. J. Clin. Nutr..

[77] Joven J., Micol V., Segura-Carretero A., Alonso-Villaverde C., Menéndez J.A. (2014). Polyphenols and the modulation of gene expression pathways: Can we eat our way out of the danger of chronic disease?. Crit. Rev. Food Sci. Nutr..

[78] Deshmukh A.B., Patel J.K. (2023). Anxiety, Gut Microbiome, and Nutraceuticals..

[79] Sudheer S., Gangwar P., Usmani Z., Sharma M., Sharma V.K., Sana S.S., Almeida F., Dubey N.K., Singh D.P., Dilbaghi N., Khayat Kashani H.R., Gupta V.K., Singh B.N., Khayatkashani M., Nabavi S.M. (2022). Shaping the gut microbiota by bioactive phytochemicals: An emerging approach for the prevention and treatment of human diseases.. Biochimie.

[80] Xu Q.Q., Su Z.R., Yang W., Zhong M., Xian Y.F., Lin Z.X. (2023). Patchouli alcohol attenuates the cognitive deficits in a transgenic mouse model of alzheimer’s disease via modulating neuropathology and gut microbiota through suppressing c/ebpβ/aep pathway.. J. Neuroinflammation.

[81] Wang X., Sun G., Feng T., Zhang J., Huang X., Wang T., Xie Z., Chu X., Yang J., Wang H., Chang S., Gong Y., Ruan L., Zhang G., Yan S., Lian W., Du C., Yang D., Zhang Q., Lin F., Liu J., Zhang H., Ge C., Xiao S., Ding J., Geng M. (2019). Sodium oligomannate therapeutically remodels gut microbiota and suppresses gut bacterial amino acids-shaped neuroinflammation to inhibit alzheimer’s disease progression.. Cell Res..

[82] Zhu F., Li C., Chu F., Tian X., Zhu J. (2020). Target dysbiosis of gut microbes as a future therapeutic manipulation in alzheimer’s disease.. Front. Aging Neurosci..

[83] Zhu G., Zhao J., Zhang H., Chen W., Wang G. (2021). Administration of bifidobacterium breve improves the brain function of aβ1-42-treated mice via the modulation of the gut microbiome.. Nutrients.

[84] Zhu G., Guo M., Zhao J., Zhang H., Wang G., Chen W. (2022). bifidobacterium breve intervention combined with environmental enrichment alleviates cognitive impairment by regulating the gut microbiota and microbial metabolites in alzheimer’s disease mice.. Front. Immunol..

[85] Bonfili L., Gong C., Lombardi F., Cifone M.G., Eleuteri A.M. (2021). Strategic modification of gut microbiota through oral bacteriotherapy influences hypoxia inducible factor-1α: Therapeutic implication in alzheimer’s disease.. Int. J. Mol. Sci..

[86] Toups K., Hathaway A., Gordon D., Chung H., Raji C., Boyd A., Hill B.D., Hausman-Cohen S., Attarha M., Chwa W.J., Jarrett M., Bredesen D.E. (2022). Precision medicine approach to alzheimer’s disease: Successful pilot project.. J. Alzheimers Dis..

[87] Qu C., Li Q.P., Su Z.R., Ip S.P., Yuan Q.J., Xie Y.L., Xu Q.Q., Yang W., Huang Y.F., Xian Y.F., Lin Z.X. (2022). Nano-honokiol ameliorates the cognitive deficits in tgcrnd8 mice of alzheimer’s disease via inhibiting neuropathology and modulating gut microbiota.. J. Adv. Res..

[88] Ma Y.Y., Li X., Yu J.T., Wang Y.J. (2024). Therapeutics for neurodegenerative diseases by targeting the gut microbiome: From bench to bedside.. Transl. Neurodegener..

[89] Zhang S., Lu J., Jin Z., Xu H., Zhang D., Chen J., Wang J. (2024). Gut microbiota metabolites: Potential therapeutic targets for alzheimer’s disease?. Front. Pharmacol..

[90] Levine B.H., Hoffman J.M. (2023). Gut microbiome transplants and their health impacts across species.. Microorganisms.

[91] Altayyar M., Nasser J.A., Thomopoulos D., Bruneau M. (2022). The implication of physiological ketosis on the cognitive brain: A narrative review.. Nutrients.

[92] Gong Z., Huang J., Xu B., Ou Z., Zhang L., Lin X., Ye X., Kong X., Long D., Sun X., He X., Xu L., Li Q., Xuan A. (2019). Urolithin a attenuates memory impairment and neuroinflammation in app/ps1 mice.. J. Neuroinflammation.

[93] Ma X., Kim J.K., Shin Y.J., Son Y.H., Lee D.Y., Park H.S., Kim D.H. (2023). Alleviation of cognitive impairment-like behaviors, neuroinflammation, colitis, and gut dysbiosis in 5xfad transgenic and aged mice by lactobacillus mucosae and bifidobacterium longum.. Nutrients.

[94] El Sayed N.S., Kandil E.A., Ghoneum M.H. (2021). Probiotics fermentation technology, a novel kefir product, ameliorates cognitive impairment in streptozotocin‐induced sporadic alzheimer’s disease in mice.. Oxid. Med. Cell. Longev..

[95] Muraleedharan A., Ray S.K. (2024). Epigallocatechin-3-gallate and genistein for decreasing gut dysbiosis, inhibiting inflammasomes, and aiding autophagy in alzheimer’s disease.. Brain Sci..

[96] Helgudóttir S.S., Mørkholt A.S., Lichota J., Bruun-Nyzell P., Andersen M.C., Kristensen N.M.J., Johansen A.K., Zinn M.R., Jensdóttir H.M., Nieland J.D.V. (2024). Rethinking neurodegenerative diseases: Neurometabolic concept linking lipid oxidation to diseases in the central nervous system.. Neural Regen. Res..

[97] Rai S.N., Singh C., Singh A., Singh M.P., Singh B.K. (2020). Mitochondrial dysfunction: A potential therapeutic target to treat alzheimer’s disease.. Mol. Neurobiol..

[98] Bourdeau-Julien I., Castonguay-Paradis S., Rochefort G., Perron J., Lamarche B., Flamand N., Di Marzo V., Veilleux A., Raymond F. (2023). The diet rapidly and differentially affects the gut microbiota and host lipid mediators in a healthy population.. Microbiome.

[99] Philip Mani A., Balasubramanian B., Mali L.A., Joseph K.S., Meyyazhagan A., Pappuswamy M., Joseph B.V. (2024). The role of the gut microbiota in neurodegenerative diseases.. Microbiol. Res. (Pavia).

[100] Persson T., Popescu B.O., Cedazo-Minguez A. (2014). Oxidative stress in alzheimer’s disease: Why did antioxidant therapy fail?. Oxid. Med. Cell. Longev..

[101] Zheng Y., Bonfili L., Wei T., Eleuteri A.M. (2023). Understanding the gut–brain axis and its therapeutic implications for neurodegenerative disorders.. Nutrients.

[102] Liu W.C., Pushparaj K., Meyyazhagan A., Arumugam V.A., Pappuswamy M., Bhotla H.K., Baskaran R., Issara U., Balasubramanian B., Mousavi Khaneghah A. (2022). Ochratoxin a as an alarming health threat for livestock and human: A review on molecular interactions, mechanism of toxicity, detection, detoxification, and dietary prophylaxis.. Toxicon.

[103] Schwerdt G., Kopf M., Gekle M. (2023). The nephrotoxin ochratoxin a impairs resilience of energy homeostasis of human proximal tubule cells.. Mycotoxin Res..

[104] Muñoz K., Cramer B., Dopstadt J., Humpf H.U., Degen G.H. (2017). Evidence of ochratoxin a conjugates in urine samples from infants and adults.. Mycotoxin Res..

[105] Ben Miri Y., Benabdallah A., Chentir I., Djenane D., Luvisi A., De Bellis L. (2024). Comprehensive insights into ochratoxin a: Occurrence, analysis, and control strategies.. Foods.

[106] Lee H.J., Kim H.D., Ryu D. (2024). Practical strategies to reduce ochratoxin a in foods.. Toxins (Basel).

[107] Mazur-Kuśnirek M., Lipiński K., Antoszkiewicz Z., Śliżewska K. (2024). The effect of synbiotics and probiotics on ochratoxin concentrations in blood and tissues, health status, and gastrointestinal function in turkeys fed diets contaminated with ochratoxin a.. Animals (Basel).

[108] Hathout A.S., Aly S.E. (2014). Biological detoxification of mycotoxins: A review.. Ann. Microbiol..

[109] Shin H.S., Lee H.J., Pyo M.C., Ryu D., Lee K.W. (2019). Ochratoxin a-induced hepatotoxicity through phase i and phase ii reactions regulated by ahr in liver cells.. Toxins (Basel).

[110] Więckowska M., Cichon N., Szelenberger R., Gorniak L., Bijak M. (2024). Ochratoxin a and its role in cancer development: A comprehensive review.. Cancers (Basel).

[111] Son Y., Lee H.J., Ryu D., Kim J.R., Kim H.Y. (2024). Ochratoxin a induces hepatic and renal toxicity in mice through increased oxidative stress, mitochondrial damage, and multiple cell death mechanisms.. Arch. Toxicol..

[112] Niaz K., Shah S.Z.A., Khan F., Bule M. (2020). Ochratoxin a–induced genotoxic and epigenetic mechanisms lead to alzheimer disease: Its modulation with strategies.. Environ. Sci. Pollut. Res. Int..

[113] Zhao P., Feng L., Jiang W., Wu P., Liu Y., Ren H., Jin X., Zhang L., Mi H., Zhou X. (2024). Unveiling the emerging role of curcumin to alleviate ochratoxin a-induced muscle toxicity in grass carp (ctenopharyngodon idella): In vitro and in vivo studies.. J. Anim. Sci. Biotechnol..

[114] Sorrenti V., Di Giacomo C., Acquaviva R., Barbagallo I., Bognanno M., Galvano F. (2013). Toxicity of ochratoxin a and its modulation by antioxidants: A review.. Toxins (Basel).

[115] Mateo E., Tonino R.P.B., Canto A., Monroy Noyola A., Miranda M., Soria J.M., Garcia Esparza M.A. (2022). The neurotoxic effect of ochratoxin-a on the hippocampal neurogenic niche of adult mouse brain.. Toxins (Basel).

[116] Li S., Zhao L., Xiao J., Guo Y., Fu R., Zhang Y., Xu S. (2024). The gut microbiome: An important role in neurodegenerative diseases and their therapeutic advances.. Mol. Cell. Biochem..

[117] Caballero-Villarraso J., Galvan A., Escribano B.M., Tunez I. (2017). Interrelationships among gut microbiota and host: Paradigms, role in neurodegenerative diseases and future prospects.. CNS Neurol. Disord. Drug Targets.

[118] Pluta R., Ułamek-Kozioł M., Januszewski S., Czuczwar S.J. (2020). Gut microbiota and pro/prebiotics in alzheimer’s disease.. Aging (Albany NY).

[119] Shankar J. (2021). Food habit associated mycobiota composition and their impact on human health.. Front. Nutr..

[120] Pisa D., Alonso R., Rábano A., Rodal I., Carrasco L. (2015). Different brain regions are infected with fungi in alzheimer’s disease.. Sci. Rep..

[121] Xu F., Fu Y., Sun T., Jiang Z., Miao Z., Shuai M., Gou W., Ling C., Yang J., Wang J., Chen Y., Zheng J.S. (2020). The interplay between host genetics and the gut microbiome reveals common and distinct microbiome features for complex human diseases.. Microbiome.

